# The Monoamine–Glutamate Continuum of Depression: A Neurobiological Framework for Precision Psychiatry

**DOI:** 10.3390/ph19050662

**Published:** 2026-04-24

**Authors:** Pietro Carmellini, Alessandro Cuomo, Maria Beatrice Rescalli, Mario Pinzi, Afendra Dourmas, Andrea Fagiolini

**Affiliations:** Department of Molecular and Developmental Medicine, Division of Psychiatry, School of Medicine, University of Siena, 53100 Siena, Italy; alessandrocuomo86@gmail.com (A.C.); beatrice.rescalli@gmail.com (M.B.R.); mario.pinzi@student.unisi.it (M.P.); fedradou@gmail.com (A.D.); andreafagiolini@gmail.com (A.F.)

**Keywords:** major depressive disorder, monoamine hypothesis, glutamate neurotransmission, synaptic plasticity, rapid-acting antidepressants, ketamine, neurobiological subtypes, precision psychiatry, biomarkers

## Abstract

**Background/Objectives**: Major depressive disorder (MDD) remains a leading cause of disability worldwide and exhibits substantial biological heterogeneity that is not adequately captured by current symptom-based diagnostic systems. While the classical monoamine hypothesis has historically guided antidepressant development, it does not fully account for variability in treatment response, delayed therapeutic onset, or the persistence of cognitive and anhedonic symptoms. Converging evidence from molecular, neuroimaging, and translational studies increasingly implicates glutamatergic dysregulation and impaired neuroplasticity as key mechanisms in depressive pathology. This narrative review aims to integrate monoaminergic and glutamatergic perspectives within a dimensional framework that may help explain clinical heterogeneity and inform mechanism-based treatment strategies. **Methods**: A narrative synthesis of the literature was conducted using major biomedical databases including PubMed, Scopus, and Web of Science. Preclinical studies, neuroimaging investigations, biomarker research, randomized clinical trials, and meta-analyses examining monoaminergic dysfunction, glutamatergic signaling, neuroplasticity pathways, and rapid-acting antidepressants were reviewed and thematically integrated. **Results**: Evidence indicates that depressive syndromes may reflect varying contributions of monoaminergic dysregulation and glutamatergic–neuroplastic impairment. Monoaminergic disturbances interact with inflammatory and neuroendocrine processes, including cytokine-driven activation of the kynurenine pathway. In parallel, alterations in glutamatergic signaling, glial function, and BDNF–TrkB–mTOR pathways contribute to synaptic atrophy and network dysfunction. Rapid-acting antidepressants such as ketamine, esketamine, and dextromethorphan–bupropion provide clinical proof-of-concept that direct engagement of synaptic plasticity mechanisms can accelerate symptom improvement, particularly in treatment-resistant depression. **Conclusions**: Integrating monoaminergic and glutamatergic mechanisms within a “monoamine–glutamate continuum” offers a conceptual framework for understanding depressive heterogeneity and treatment response. Multimodal approaches combining clinical phenotyping with inflammatory, neuroimaging, and molecular markers may ultimately support mechanism-informed precision psychiatry strategies in major depressive disorder.

## 1. Introduction

Major depressive disorder (MDD) remains one of the most disabling psychiatric illnesses worldwide, characterized by chronicity, frequent relapse, and medical comorbidity. Despite major advances in neuroscience, its diagnostic construct continues to rely on symptom clusters rather than underlying biological mechanisms. The prevailing nosological systems (DSM-5, ICD-11) group patients with heterogeneous etiologies and neurobiology under a unified diagnostic rubric, thereby diluting the capacity to stratify patients by mechanistic pathways or to predict differential response [1].

Clinically, this heterogeneity is manifested in widely variable treatment outcomes: a substantial proportion of patients do not achieve full remission with first-line monoaminergic agents, many retain residual symptoms in domains such as cognition, anhedonia, or motivation, and a nontrivial subset progresses into treatment-resistant depression (TRD). Compounding this is the well-known latency of response to conventional antidepressants, typically requiring weeks to months, suggesting that these agents act primarily via downstream adaptive processes rather than directly correcting upstream pathological disturbances [2]. For decades, the monoamine hypothesis, postulating that dysregulation or deficiency in serotonergic, noradrenergic, and dopaminergic signaling underlies depressive symptomatology, has provided the conceptual scaffold for antidepressant drug development [3,4]. Indeed, early clinical observations (e.g., responses to tricyclic antidepressants and monoamine oxidase inhibitors) reinforced the notion that boosting monoamine transmission could alleviate mood symptoms. However, accumulating evidence challenges the sufficiency of this model: monoamine depletion paradigms fail to consistently induce depressive symptoms in healthy individuals, many patients relapse despite sustained serotonergic or noradrenergic treatment, and augmentation or neuromodulatory strategies, such as lithium, thyroid hormone, or electroconvulsive therapy, exert effects beyond simple monoamine potentiation [5,6]. Thus, while monoaminergic dysregulation likely contributes to depression pathophysiology in many individuals, it does not fully account for the breadth of clinical and neurobiological observations [7].

In recent years, increasing evidence has underscored the role of glutamatergic imbalance and impaired neuroplasticity in the pathophysiology of depression [1,8,9]. Altered glutamate metabolism and reduced neurotrophic signaling converge on synaptic atrophy and connectivity loss, bridging stress, inflammation, and neuronal dysfunction [10,11,12,13]. The advent of ketamine as a rapid-acting antidepressant has served as a critical proof of concept that modulation of synaptic plasticity can yield fast and clinically meaningful mood improvements, even in TRD populations [14,15]. Clinical observations suggest that biological heterogeneity is thought to contribute to differential treatment response: while patients with prominent HPA-axis dysregulation or melancholic features may demonstrate comparatively better response patterns to dual-action monoaminergic strategies in some studies, although effect sizes vary, those with features suggestive of impaired neuroplasticity, such as TRD or inflammatory presentations, may be more likely to benefit from glutamatergic interventions, although this remains an evolving area of investigation [9,16,17].

**Figure 1:**
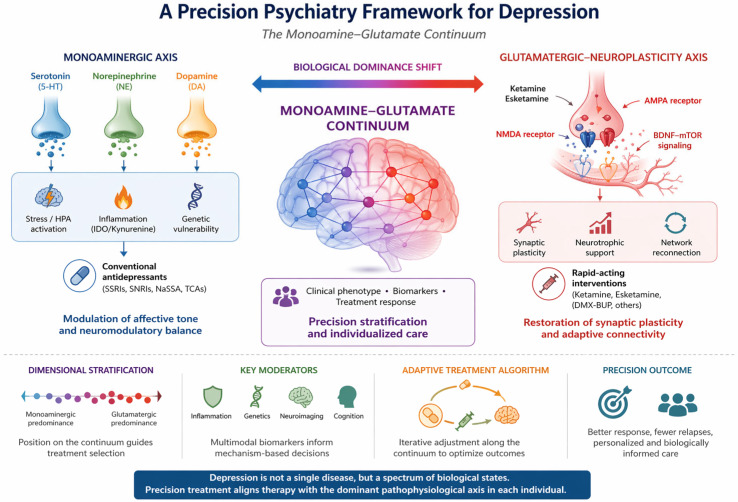
This schematic representation illustrates the proposed monoamine–glutamate continuum in major depressive disorder, conceptualized as a dynamic shift in biological dominance across systems. On the left, monoaminergic dysregulation—modulated by stress-related HPA axis activation, inflammatory pathways (e.g., IDO/kynurenine), and genetic vulnerability—affects serotonergic, noradrenergic, and dopaminergic neurotransmission and is primarily targeted by conventional antidepressants. On the right, glutamatergic dysfunction is associated with impaired synaptic plasticity and network connectivity, involving NMDA and AMPA receptor dynamics as well as BDNF–mTOR signaling, and is targeted by rapid-acting interventions such as ketamine and esketamine. The central continuum reflects a dimensional framework in which the relative contribution of monoaminergic versus glutamatergic mechanisms shapes clinical phenotype, biomarker profiles, and treatment response. This model supports precision psychiatry approaches based on stratification along the continuum and iterative, mechanism-informed treatment adaptation.

Emerging biomarker modalities, such as 1H-MRS glutamate/Glx metrics, functional connectivity and network integrity, EEG indices, inflammatory cytokines, and BDNF/TrkB genetic or epigenetic variation, hold promise for predicting differential treatment response and guiding mechanism-informed stratification [10,18]. Despite these advances, current diagnostic frameworks remain largely phenomenological, clustering biologically diverse entities under a single descriptive label and obscuring circuit-level heterogeneity. The NIMH’s Research Domain Criteria (RDoC) initiative and the Hierarchical Taxonomy of Psychopathology (HiTOP) framework both advocate dimensional, data-driven approaches that bridge clinical observation with biological measurement [19,20]. Integrating these paradigms into depression research may accelerate the identification of neurobiologically coherent subtypes amenable to precision treatment. This review conceptualizes depression as a mechanistic continuum spanning monoaminergic dysregulation and glutamatergic–plasticity impairment. A schematic overview of the proposed monoamine–glutamate continuum and its clinical implications is provided in Figure 1. We synthesize mechanistic, translational, and clinical evidence across these biological axes to outline a framework for precision psychiatry, aiming to optimize treatment matching and reduce the trial-and-error burden inherent in current practice. In this context, the present review conceptualizes depression as a mechanistic continuum spanning monoaminergic dysregulation and glutamatergic–plasticity impairment. We first examine the monoaminergic axis and its clinical correlates, then review the glutamatergic and neuroplasticity axis, and finally integrate these domains into a dimensional framework for patient stratification, biomarker-informed interpretation, and mechanism-based treatment selection. This structured approach aims to bridge mechanistic understanding with clinical decision-making in a precision psychiatry perspective.

## 2. Materials and Methods

A comprehensive literature search was performed in PubMed, Scopus, and Web of Science databases up to September 2025. The search combined Medical Subject Headings (MeSH) and free-text terms related to monoaminergic and glutamatergic mechanisms in major depressive disorder (MDD). Keywords included: “monoamine hypothesis,” “serotonin,” “dopamine,” “norepinephrine,” “glutamate,” “NMDA receptor,” “AMPA receptor,” “BDNF–mTOR signaling,” “synaptic plasticity,” “neuroinflammation,” “HPA axis,” “rapid-acting antidepressants,” “ketamine,” “esketamine,” “dextromethorphan–bupropion,” “precision psychiatry,” “RDoC,” and “HiTOP.”

Additional sources were identified by manual screening of reference lists from relevant publications and high-impact reviews to ensure coverage of the most recent and conceptually relevant evidence.

We included peer-reviewed articles published in English encompassing both preclinical and clinical research, including animal studies, molecular and cellular investigations, neuroimaging and biomarker studies, randomized controlled trials, meta-analyses, and expert reviews addressing the biological interplay between monoaminergic dysregulation and glutamatergic–neuroplastic impairment.

Exclusion criteria were case reports, conference abstracts, non–peer-reviewed materials, and articles focusing solely on unrelated neurotransmitter systems. To minimize selective citation bias, we applied an a priori hierarchy of evidence. Meta-analyses and randomized controlled trials were prioritized when available, followed by well-powered observational studies and replicated neuroimaging or translational findings. Preclinical and mechanistic studies were included primarily when they converged with human data or provided explanatory frameworks for clinical observations.

Given the conceptual aim of this review, emphasis was placed on triangulation across domains—molecular biology, neuroimaging, inflammatory markers, and pharmacological response—rather than reliance on single-study findings.

Relevant findings were extracted and thematically organized into three domains: 1: Monoaminergic axis: molecular mechanisms, pharmacological evidence, and phenotype-specific treatment response; 2: Glutamatergic and neuroplasticity axis: synaptic and cellular mechanisms, rapid-acting antidepressants, and translational implications; 3: Cross-axis integration: interaction between monoamine and glutamate systems, biomarker-guided stratification, and clinical heuristics for precision treatment.

Given the heterogeneity of designs, outcomes, and methodologies, a narrative synthesis was conducted rather than a quantitative meta-analysis. Evidence was critically appraised for conceptual relevance, mechanistic insight, and translational validity, emphasizing studies elucidating the dynamic interplay between neurotransmission, plasticity, and clinical phenotype.

The synthesis aimed to develop a unified conceptual model, the Monoamine–Glutamate Continuum of Depression, integrating neurochemical, cellular, and network-level findings into a dimensional framework for precision subtyping.

## 3. The Monoamine-Glutamate Continuum: A Conceptual Framework

**Figure 2:**
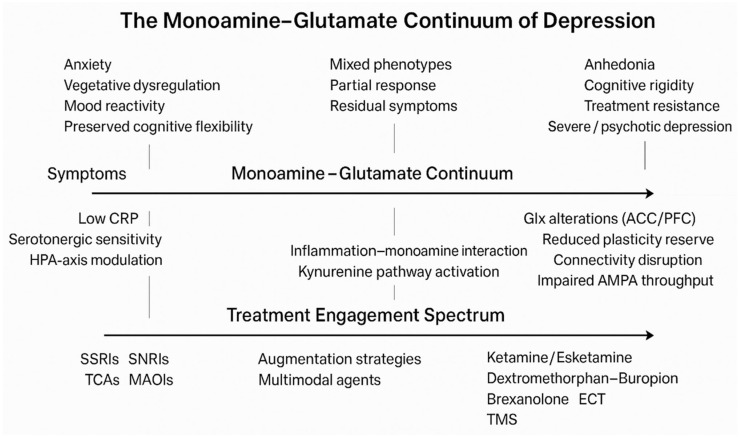
Heuristic research model, not a clinical guideline; This schematic illustrates major depressive disorder as a dimensional continuum defined by the relative predominance of monoaminergic regulatory dysfunction versus glutamatergic–neuroplastic impairment. Symptom clusters, biological anchors, and treatment mechanisms are aligned along the same axis to emphasize functional matching rather than categorical diagnosis. The model is heuristic and intended to guide research and hypothesis generation rather than serve as a prescriptive clinical algorithm. Abbreviations: 5-HT, serotonin; NE, norepinephrine; DA, dopamine; NMDA, N-methyl-D-aspartate receptor; AMPA, α-amino-3-hydroxy-5-methyl-4-isoxazolepropionic acid receptor; BDNF, brain-derived neurotrophic factor; mTOR, mechanistic target of rapamycin; SSRIs, selective serotonin reuptake inhibitors; SNRIs, serotonin–norepinephrine reuptake inhibitors.

Recent advances in neurobiology have redefined the understanding of MDD. The classical monoamine hypothesis, once dominant, no longer suffices to explain the clinical heterogeneity, chronicity, and variable treatment response that define depression. Increasingly, evidence points to two intertwined biological systems, the monoaminergic regulatory network and the glutamatergic–neuroplasticity system, as complementary contributors to the disorder. These domains can be viewed as the poles of a monoamine–glutamate continuum, a mechanistic spectrum along which depressive syndromes are distributed according to the relative prominence of neuromodulatory versus synaptic dysfunction. On one end, monoaminergic dysregulation shapes mood, stress reactivity, and reward processing through alterations in serotonin, norepinephrine, and dopamine signaling. On the other, glutamatergic imbalance and impaired plasticity lead to reduced synaptic connectivity, cognitive slowing, and treatment resistance. Rather than representing mutually exclusive models, monoaminergic and glutamatergic mechanisms are best understood as partially overlapping and biologically interacting domains that may converge on shared downstream processes, including stress adaptation, neurotrophic signaling, and circuit remodeling. Chronic stress, inflammation, and neuroendocrine alterations disrupt monoamine metabolism while simultaneously impairing glutamatergic signaling and neurotrophic support. This biological interplay may help explain part of the heterogeneity in antidepressant response across pharmacological classes, rather than implying a uniform mechanism across all antidepressants. For example, monoaminergic agents such as SSRIs, SNRIs, TCAs, and MAOIs differ substantially in pharmacodynamic profile, while rapid-acting glutamatergic agents engage synaptic plasticity through distinct and faster mechanisms. Accordingly, variability in efficacy is likely to reflect both differences between drug classes and differences in the dominant biological features of individual depressive presentations. Understanding depression as a continuum, spanning from monoaminergic regulation to glutamatergic plasticity, offers a conceptual bridge between neurochemistry, inflammation, and circuit-level remodeling. It invites a precision-based approach, in which treatment selection aligns with the prevailing neurobiological signature rather than a categorical diagnosis. The following chapters explore these axes in depth: first, the monoaminergic system, its mechanisms and phenotypic correlates; and subsequently, the glutamatergic and neuroplasticity axis, where rapid-acting antidepressants exemplify the translation of molecular insight into clinical practice. This continuum (Figure 1 and Figure 2) is intended as a heuristic, dimensional framework rather than a validated clinical taxonomy. Its clinical utility will depend on prospective biomarker-stratified trials and harmonized multimodal datasets capable of testing mechanistic predictions.

## 4. The Monoaminergic Axis

We begin by examining the monoaminergic axis, which historically represents the primary neurobiological framework for understanding depression and remains the foundation of most currently available pharmacological treatments.

### 4.1. Mechanistic Overview

While these mechanisms are well established, they are briefly summarized here to provide the biological foundation for the subsequent precision-oriented framework. Building on the monoamine hypothesis, serotonin (5-HT), norepinephrine (NE), and dopamine (DA) exert widespread modulatory control over cortical and limbic circuits, influencing emotional regulation, stress responsiveness, and reward processing via projections from the raphe nuclei, locus coeruleus, and ventral tegmental area [4,7]. Genetic variability in key transporters, including SERT (SLC6A4), NET (SLC6A2), and DAT (SLC6A3), contributes to interindividual differences in synaptic monoamine availability and stress reactivity [21,22].

Monoaminergic signaling engages intracellular pathways such as cAMP–CREB and ERK–MAPK, promoting the expression of neurotrophic factors including BDNF, thereby linking neurotransmission to synaptic plasticity [13,23,24]. Disruptions in these pathways are associated with reduced trophic support and maladaptive remodeling within cortico-limbic networks. Consistently, neuroimaging studies show altered serotonin transporter binding and dysfunctional connectivity between the subgenual anterior cingulate cortex, amygdala, and prefrontal regions, patterns that correlate with symptom severity and treatment response [25,26,27,28].

Monoaminergic dysfunction is closely intertwined with neuroendocrine and immune processes. Chronic stress induces sustained activation of the hypothalamic–pituitary–adrenal (HPA) axis, leading to elevated cortisol, impaired glucocorticoid feedback, and structural vulnerability in hippocampal and prefrontal regions [29]. In parallel, pro-inflammatory cytokines (e.g., IL-6, TNF-α, interferon-γ) activate the indoleamine-2,3-dioxygenase pathway, reducing serotonin synthesis and shifting tryptophan metabolism toward kynurenine derivatives [30]. Inflammatory signaling also suppresses dopaminergic transmission, contributing to anhedonia and motivational deficits [31].

Together, these findings suggest that monoaminergic dysfunction extends beyond simple neurotransmitter deficiency and may reflect a broader disruption of neuromodulatory homeostasis. Alterations in receptor sensitivity, intracellular signaling, and neurotrophic support contribute to structural and functional disconnection within affective circuits, which may manifest clinically as low mood, anhedonia, and cognitive impairment. This framework provides the biological basis for monoaminergic treatments, which aim not only to restore neurotransmitter balance but also to engage downstream neuroplastic and neuroendocrine processes.

### 4.2. Pharmacological Landscape and Clinical Evidence

Building upon this neurobiological foundation, pharmacological interventions targeting the monoaminergic axis have long represented the core of antidepressant therapy. The major pharmacological classes, selective serotonin reuptake inhibitors (SSRIs), serotonin–norepinephrine reuptake inhibitors (SNRIs), tricyclic antidepressants (TCAs), monoamine oxidase inhibitors (MAOIs), and newer multimodal agents, share the common goal of enhancing monoamine transmission or modulating receptor activity yet differ in their receptor affinities and downstream signaling profiles [32,33]. Large-scale meta-analyses confirm that SSRIs and SNRIs are effective in alleviating depressive symptoms compared with placebo, although the effect sizes are modest [33]. TCAs remain among the most potent antidepressants, but their clinical use is limited by anticholinergic, antihistaminergic, and cardiotoxic side effects. MAOIs, while effective particularly in atypical and refractory forms, require strict dietary restrictions due to the risk of hypertensive crises [34,35]. The development of newer agents such as vortioxetine and mirtazapine has broadened the monoaminergic paradigm by integrating receptor modulation with reuptake inhibition. Vortioxetine combines serotonin reuptake inhibition with multimodal receptor activity, 5-HT_1_A agonism, 5-HT_1_B partial agonism, and 5-HT_3_ antagonism, producing benefits across cognitive domains of depression, beyond mood improvement alone [36]. Mirtazapine, through α_2_-adrenergic blockade and 5-HT_2_/5-HT_3_ antagonism, enhances noradrenergic and serotonergic transmission and exerts anxiolytic and sedative effects, often advantageous for patients with insomnia or anxiety comorbidity [37]. Augmentation strategies have further expanded the therapeutic scope of monoamine-based treatments. Lithium remains a cornerstone of augmentation, enhancing serotonergic neurotransmission and reducing suicidality through neuroprotective and intracellular signaling mechanisms [38]. Thyroid hormone (T_3_) supplementation can accelerate antidepressant response through genomic and non-genomic pathways, particularly in partial responders [39]. Additionally, atypical antipsychotics such as aripiprazole and quetiapine XR, acting as partial dopamine D_2_ agonists and 5-HT_1_A modulators, have proven efficacious in augmenting antidepressant therapy, particularly for treatment-resistant or anxious forms of depression [40]. Neuroimaging data support the biological plausibility of these interventions. Treatment with SSRIs normalizes amygdala hyperreactivity and enhances prefrontal engagement, reflecting restoration of top-down control mechanisms [41]. Longitudinal PET studies demonstrate partial recovery of serotonin transporter binding in responders, mirroring clinical improvement [42]. Collectively, these findings underscore that antidepressants exert their effects not merely by increasing synaptic monoamines, but by initiating neuroadaptive processes that re-establish network-level homeostasis and synaptic plasticity over time.

### 4.3. Phenotypic Correlates and Moderators of Response

Despite their widespread efficacy, monoaminergic agents produce highly variable outcomes across individuals. This heterogeneity reflects the underlying biological diversity of depressive syndromes and has led to the identification of partially distinct clinical subtypes, melancholic, atypical, anxious, inflammatory, and seasonal, each associated with characteristic symptom profiles and differential treatment responses.

Patients with melancholic depression have in some studies shown comparatively stronger response patterns to dual-action antidepressants or tricyclics than to purely serotonergic agents. Objective psychomotor disturbance, quantified through the CORE scale, has been identified as the single most discriminant feature of melancholic depression, surpassing classical endogeneity symptoms in diagnostic specificity [43]. These individuals often exhibit HPA-axis hyperactivity and glucocorticoid receptor resistance, consistent with a neuroendocrine subtype in which monoaminergic and stress pathways converge. In contrast, atypical depression, characterized by mood reactivity, hypersomnia, and hyperphagia, has been associated with enhanced central sensitivity to acetylcholine and diminished dopaminergic tone. Clinically, such patients display superior response to MAOIs and, to a lesser extent, SSRIs. Meta-analytic data demonstrate that atypical depression shows a preferential response to monoamine oxidase inhibitors, particularly phenelzine, compared with tricyclic antidepressants, supporting a distinct neurobiological substrate involving dopaminergic and serotonergic imbalance [44]. This profile aligns with a pattern of monoaminergic dysregulation favoring serotonergic imbalance over noradrenergic depletion.

Anxious depression, a highly prevalent subtype affecting nearly half of MDD cases, is associated with heightened limbic reactivity and reduced cortical inhibition. It has been consistently associated with poorer treatment outcomes across pharmacological strategies, often necessitating combined serotonergic–noradrenergic approaches or augmentation with atypical antipsychotics or benzodiazepines. In the STAR*D trial, over half of patients met criteria for anxious depression, a phenotype characterized by greater illness burden, suicidality, and lower remission rates despite optimized antidepressant strategies [45]. These patients illustrate the overlap between affective and anxiety circuits, where monoaminergic interventions may require reinforcement by GABAergic or dopaminergic modulation. Seasonal affective disorder (SAD) occupies a unique position along the monoaminergic spectrum, representing a state of recurrent depressive episodes triggered by reduced photoperiodicity and subsequent alterations in serotonergic and circadian signaling. Light therapy and prophylactic bupropion XL are well-established preventive options [46]. Importantly, a clinical trial demonstrated that sertraline can effectively prevent depressive recurrence in SAD, reinforcing the central role of serotonergic vulnerability in its pathophysiology [47]. This finding underscores how seasonal modulation of serotonergic tone interacts with circadian and environmental factors, further linking SAD to the broader monoamine-based framework of depression.

Finally, a growing body of evidence defines inflammatory depression as a biologically distinct phenotype characterized by fatigue, anhedonia, psychomotor slowing, and cognitive dulling, often accompanied by elevated inflammatory biomarkers such as CRP and IL-6 [48]. Elevated inflammatory burden has been associated with lower response rates to serotonergic antidepressants and, in some studies, comparatively better outcomes with noradrenergic or tricyclic agents, suggesting that inflammation-induced IDO activation and altered tryptophan metabolism directly compromise serotonergic signaling. This pattern integrates immune activation with monoaminergic imbalance, reflecting a shift toward the noradrenergic end of the spectrum. Beyond categorical subtypes, inflammation also emerges as a key biological moderator of antidepressant response. Baseline CRP levels predict pharmacologic sensitivity: lower CRP favors SSRI efficacy, whereas elevated CRP identifies patients more likely to respond to noradrenergic or tricyclic agents [49]. Other moderators include genetic variations in the serotonin transporter promoter (5-HTTLPR), which modulate susceptibility to stress and influence treatment outcomes, as well as neuroimaging correlates, heightened amygdala activation predicting SSRI response, and dorsolateral prefrontal hypoactivity or disrupted subgenual ACC connectivity signaling treatment resistance [21,27,50].

Together, these data illustrate that the monoaminergic axis encompasses a rich diversity of mechanistic and clinical expressions. Recognizing these subtypes and moderators refines therapeutic selection and supports a more personalized approach to treatment.

These clinical patterns and moderators of response are summarized in Table 1.

## 5. The Glutamatergic/Synaptic Plasticity Axis

In contrast to the monoaminergic framework, the glutamatergic and neuroplasticity axis emphasizes fast-acting synaptic mechanisms and circuit-level remodeling as key drivers of depressive symptomatology and treatment response.

### 5.1. Mechanistic Overview

In parallel with monoaminergic dysregulation, converging evidence implicates glutamatergic dysfunction and impaired synaptic plasticity as key contributing mechanisms of major depressive disorder. Glutamate is the primary excitatory neurotransmitter in the central nervous system, orchestrating synaptic communication, cortical oscillations, and activity-dependent neuroplasticity [1]. Chronic stress, neuroinflammation, and metabolic alterations disrupt glutamate homeostasis through excessive presynaptic release, reduced astrocytic uptake, and impaired vesicular cycling [51].

Postmortem and magnetic resonance spectroscopy (1H-MRS) studies reveal decreased glutamate and Glx levels in the prefrontal cortex and anterior cingulate cortex (ACC) of patients with MDD. These findings indicate a region-specific imbalance, with cortical hypoglutamatergia and limbic hyperglutamatergia contributing to mood dysregulation and cognitive inflexibility [10]. At the receptor level, NMDA receptor dysregulation, particularly involving NR2A and NR2B subunits, may contribute to excitotoxic signaling and downstream impairment of AMPA throughput [52]. Reduced activation of the BDNF–TrkB–mTOR axis decreases synaptogenesis and dendritic spine maintenance, linking molecular deficits to structural brain changes observed in depression [53].

Stress-induced activation of the kynurenine pathway further amplifies glutamatergic dysfunction: conversion of tryptophan to quinolinic acid enhances NMDA receptor stimulation and oxidative stress, thereby integrating inflammatory signaling with excitotoxic injury [54]. This neuroimmune interface provides a mechanistic explanation for inflammatory subtypes of depression characterized by anhedonia and psychomotor slowing. Functional imaging consistently demonstrates disrupted default mode network (DMN) and salience network connectivity in depressed patients, often normalizing with glutamatergic or neurotrophic treatments [55]. Electrophysiological studies have linked ketamine-induced increases in gamma oscillations to enhanced synaptic plasticity, serving as a potential biomarker of treatment engagement [56]. Overall, these findings converge on a model where glutamatergic dysregulation, mediated by NMDA receptor overactivity, AMPA receptor hypoactivity, and BDNF–mTOR suppression, leads to structural and functional synaptic deficits. Restoration of glutamatergic balance thus represents a key therapeutic mechanism distinct from, yet complementary to, monoaminergic modulation.

Beyond neuronal mechanisms, glial dysfunction represents a crucial determinant of glutamatergic imbalance. Astrocytes regulate extracellular glutamate through excitatory amino acid transporters EAAT1 and EAAT2; down-regulation of these transporters under chronic stress or inflammation leads to impaired clearance and excitotoxic injury [57]. Microglial activation further amplifies this dysregulation through the kynurenine pathway: inflammatory cytokines upregulate IDO and kynurenine-3-monooxygenase, increasing neurotoxic quinolinic acid, an NMDA receptor agonist, while reducing the neuroprotective metabolite kynurenic acid [58]. The resulting quinolinic/kynurenic acid imbalance constitutes a measurable biochemical signature of inflammatory depression and correlates with reduced cortical glutamate turnover and treatment resistance [59].

### 5.2. Pharmacological Landscape and Clinical Evidence

Clinical exploitation of the glutamatergic axis began with the serendipitous discovery of ketamine’s rapid antidepressant effects. A single intravenous infusion of subanesthetic ketamine (0.5 mg/kg) has been shown to induce rapid and clinically meaningful symptom improvement within hours, even in treatment-resistant depression (TRD) [15]. Multiple controlled studies and meta-analyses have confirmed these rapid, albeit transient, effects [60]. Mechanistically, ketamine antagonizes NMDA receptors on inhibitory interneurons, causing disinhibition of glutamatergic pyramidal neurons and a transient surge in synaptic glutamate. This is thought to involve AMPA receptor activation, enhances BDNF release, and triggers mTORC1-dependent synaptogenesis. Within hours, ketamine increases dendritic spine density and restores prefrontal connectivity [53]. The S-enantiomer esketamine, approved by the FDA and EMA for TRD, demonstrates comparable efficacy with a more favorable safety profile when administered intranasally [61]. Esketamine has been associated with clinically significant symptom reduction within 24 h and maintains its effect when combined with oral antidepressants [62]. The dextromethorphan–bupropion combination, an oral NMDA receptor modulator with sigma-1 receptor agonism and monoaminergic reuptake inhibition, has shown rapid-acting antidepressant properties with favorable tolerability [63]. Its dual action bridges the monoaminergic and glutamatergic systems, supporting the conceptual continuum between the two axes. Adjunctive or experimental glutamatergic agents, including lamotrigine, riluzole, N-acetylcysteine (NAC), and investigational compounds such as rapastinel and scopolamine, have shown variable results, warranting further study [8]. These compounds, though mechanistically diverse, converge on the enhancement of glutamatergic throughput or facilitation of synaptic plasticity. Beyond pharmacology, neurosteroid modulators such as brexanolone (intravenous allopregnanolone) and zuranolone (oral analogue) represent another class of rapid-acting agents operating on GABA-A receptor subtypes that indirectly normalize excitatory–inhibitory balance. Brexanolone’s efficacy in postpartum depression underscores the relevance of plasticity modulation across etiologically distinct depressive states [64]. Rapid-acting antidepressants provide strong clinical proof-of-concept that engaging synaptic plasticity can accelerate symptom relief; however, mechanistic mediation in humans remains only partially established and is often inferred from convergent biomarker, imaging, and translational evidence

### 5.3. Evidence-Linked Clinical Phenotypes

Converging lines of evidence indicate that dysfunctions in glutamatergic signaling and neuroplastic remodeling constitute a shared substrate across several clinical variants of major depressive disorder. Alterations in synaptic potentiation, dendritic remodeling, and excitatory–inhibitory homeostasis appear to determine the persistence, phenomenology, and treatment responsiveness of depressive states. Within this conceptual framework, the glutamatergic/neuroplasticity axis emerges as a transdiagnostic mechanism capable of integrating the biological and phenomenological heterogeneity of depression. The most direct evidence derives from treatment-resistant depression (TRD), where conventional monoaminergic antidepressants fail to induce neuroadaptive changes within cortico–limbic networks. In these patients, NMDA receptor antagonists such as ketamine and its S-enantiomer esketamine, produce a rapid, dose-dependent antidepressant effect through transient inhibition of GABAergic interneurons, generating a glutamate surge and downstream AMPA receptor activation that triggers mTOR-dependent synaptogenesis [15]. Neuroimaging studies demonstrate a parallel normalization of prefrontal global brain connectivity (GBCr), suggesting that clinical remission is mediated by restoration of functional network integrity rather than neurotransmitter replenishment [16]. This evidence supports the interpretation that TRD may involve impaired neuroplastic capacity, though this construct remains theoretical and under empirical refinement, one that can be reversed by interventions capable of reopening windows of synaptic adaptability. Comparable mechanisms have been identified in bipolar depression, where episodes of persistent low mood and cognitive rigidity are similarly linked to disrupted glutamatergic tone. A single subanesthetic infusion of ketamine elicits a rapid and robust improvement in mood and affective reactivity, even in individuals receiving mood stabilizers [65]. The convergence of findings across unipolar and bipolar depression suggests that treatment resistance reflects a common neurobiological endpoint characterized by synaptic disconnection and reduced capacity for dendritic renewal, positioning glutamate-mediated plasticity as a transdiagnostic therapeutic target. In postpartum depression (PPD), the pathophysiological mechanism involves an abrupt reduction in neurosteroid-mediated inhibition following childbirth. The decline in allopregnanolone concentration leads to decreased GABA_A receptor sensitivity and heightened cortical excitability, resulting in mood instability and affective dysregulation. Brexanolone, an intravenous formulation of synthetic allopregnanolone, acts as a positive allosteric modulator of both synaptic and extrasynaptic GABA_A receptors, rapidly restoring inhibitory tone and producing sustained symptom remission [64]. This mechanism illustrates how disturbances in the excitatory–inhibitory balance can converge on the same neuroplastic pathway that underlies glutamatergic dysfunction, reinforcing the notion that restoration of network homeostasis is central to affective recovery. Late-life and psychotic depression further exemplify the interface between clinical phenotype and large-scale network remodeling. These severe forms are often associated with structural and functional disconnection within hippocampal–limbic circuits, resulting in cognitive impairment, psychomotor retardation, and emotional blunting. Electroconvulsive therapy (ECT) remains uniquely effective in these presentations, and longitudinal neuroimaging studies reveal transient hippocampal and amygdalar volume increases following treatment, independent of serum BDNF levels [66]. Such findings point to a process of macroplastic reorganization, possibly reflecting angiogenesis, neurogenesis, and synaptogenesis, that reinstates connectivity within disrupted affective circuits. The temporal dissociation between volumetric changes and symptom improvement suggests that ECT activates a multiphasic cascade of neuroplastic processes operating beyond immediate clinical response. Anhedonia, defined by loss of reward sensitivity and motivational drive, represents a functional endpoint of impaired synaptic potentiation. This dimension captures a core feature of depression that directly reflects the state of glutamatergic throughput and AMPA receptor activity. Both preclinical and clinical data demonstrate that rapid-acting agents capable of enhancing excitatory signaling can restore reward-related behavior and motivational engagement. The ketamine metabolite (2R,6R)-hydroxynorketamine (HNK) exerts robust antidepressant-like effects through AMPA receptor activation and BDNF release, independent of NMDA blockade, and without dissociative or psychotomimetic properties [14]. Similarly, the oral combination dextromethorphan–bupropion, acting as an NMDA antagonist and sigma-1 receptor agonist, enhances glutamatergic transmission and produces significant and sustained improvements in depressive and anhedonic symptoms [63]. Together, these findings emphasize that restoration of synaptic efficiency is considered a key mechanism underlying the rapid and sustained remission observed with glutamatergic modulators. Overall, these clinical dimensions delineate depression may be conceptualized, in part, as a disorder of impaired neuroplastic adaptability rather than neurotransmitter deficiency. Variants such as treatment-resistant, bipolar, postpartum, psychotic, and anhedonic depression represent distinct expressions of the same disrupted plasticity continuum. Across these phenotypes, interventions targeting the glutamatergic/neuroplasticity axis, whether through NMDA antagonism, AMPA potentiation, or modulation of inhibitory tone, restore the brain’s capacity for adaptive remodeling and functional integration. This unified framework supports the emerging paradigm of precision psychiatry, in which therapeutic strategies are aligned with neurobiological signatures of plasticity failure rather than categorical diagnosis. These phenotypic expressions linked to glutamatergic and neuroplastic dysfunction are summarized in Table 2. The integration of monoaminergic, inflammatory, and glutamatergic mechanisms into a unified continuum model is illustrated in Figure 1.

## 6. Cross-Axis Integration and Patient Stratification

### 6.1. Molecular Divergence as Clinical Opportunity

The classical monoamine hypothesis of depression, first articulated by Schildkraut in “The Catecholamine Hypothesis of Affective Disorders”, proposed that mood disorders arise from deficits in central catecholamine function [67]. This view, later extended to serotonergic systems, shaped decades of antidepressant development [68]. While it provided a coherent neurochemical model and guided the introduction of tricyclics and SSRIs, its limitations soon became evident: therapeutic latency, partial response, and the persistence of cognitive and motivational deficits despite monoaminergic normalization [4,7]. Over the past two decades, a broader integrative framework has emerged, positioning glutamatergic dysfunction and impaired neuroplasticity as core drivers of depressive pathology [1]. In this perspective, monoamines act as modulators of neural tone and network stability, while glutamate orchestrates synaptic adaptability, dendritic remodeling, and circuit-level integration [13]. Depression thus reflects a failure of coordination between these systems, a collapse of homeostatic signaling that undermines resilience and prevents adaptive reconnection following stress or trauma [69]. Experimental findings have clarified that monoaminergic and glutamatergic systems are deeply interdependent. Serotonergic and dopaminergic projections regulate glutamate release in cortico–limbic circuits, whereas glutamatergic activity shapes the firing dynamics of midbrain monoaminergic neurons [70]. Moreover, interventions that potentiate synaptic plasticity, such as ketamine, electroconvulsive therapy, and neurosteroid treatments, often produce secondary normalization of monoaminergic tone, underscoring the reciprocal coupling between neurochemical transmission and structural remodeling [2,23]. From this integrated viewpoint, monoamine- and glutamate-based treatments represent distinct but complementary levels of engagement within a unified plasticity continuum. Monoaminergic agents act as slow facilitators of synaptic readiness, stabilizing network tone, whereas glutamatergic modulators directly reopen windows of plastic potential, accelerating synaptogenesis and network recalibration [15,16]. This synthesis reframes depression not as a static neurotransmitter deficiency but as a dynamic disorder of impaired adaptive connectivity, where recovery depends on re-establishing synaptic plasticity and network integration.

### 6.2. Practical Heuristics for Stratification

The integration of monoaminergic and glutamatergic frameworks yields a practical heuristic for understanding and stratifying depressive presentations. Rather than discrete disease categories, major depressive states can be positioned along a biological continuum defined by the relative predominance of neurotransmitter modulation versus neuroplastic remodeling [1].

In this perspective, the monoaminergic axis regulates affective tone, motivation, and psychomotor energy through serotonergic, noradrenergic, and dopaminergic systems, whereas the glutamatergic–plasticity axis mediates adaptive connectivity, cognitive flexibility, and the capacity for experience-dependent reorganization [13,71]. From a clinical standpoint, this conceptual model allows the use of functional heuristics to anticipate treatment response and guide mechanistic targeting.

Patients whose symptomatology is dominated by low mood, anxiety, and vegetative dysregulation, with preserved cognitive flexibility, often correspond to a monoaminergic-dominant subtype, more likely to respond to serotonergic or noradrenergic antidepressants [4]. Conversely, anhedonic, ruminative, and treatment-resistant profiles, typically marked by motivational blunting and cognitive rigidity, reflect glutamatergic and neuroplasticity deficits, responding preferentially to agents that directly modulate synaptic potentiation, such as ketamine, esketamine, and dextromethorphan–bupropion [2,15]. This heuristic can also incorporate neurobiological markers of plasticity reserve, such as prefrontal connectivity indices, BDNF levels, and cognitive flexibility scores, which correlate with the brain’s ability to mount structural adaptation [16,69]. Patients with preserved connectivity and stress resilience may benefit from gradual monoaminergic modulation, while those with network rigidity or neurotrophic depletion may require interventions that reopen windows of plastic potential [72]. Importantly, these heuristics are not intended as rigid categories but as dimensional guides for personalized treatment. They operationalize the biological continuum outlined in Table 2, translating molecular and circuit-level insights into clinical decision points. By conceptualizing depression in terms of dominant pathophysiological axes rather than syndromic labels, clinicians can select interventions that align with each patient’s neurobiological profile, bridging the gap between mechanism and phenotype. In recent years, renewed interest has emerged around serotonergic psychedelics, such as psilocybin and LSD, as potential therapeutic agents in depression. These compounds primarily act as 5-HT2A receptor agonists, inducing transient states of enhanced neural entropy and increased functional connectivity across large-scale brain networks. Importantly, their effects appear to extend beyond acute perceptual changes, promoting enduring increases in synaptic plasticity, emotional processing, and cognitive flexibility. From a mechanistic perspective, psychedelics may represent an additional entry point within the monoamine–glutamate continuum, where serotonergic receptor activation leads to downstream glutamatergic modulation and neuroplastic reorganization. This positions psychedelic-assisted therapy as a potential precision-oriented intervention, particularly in patients characterized by rigid cognitive patterns, maladaptive network dynamics, and treatment resistance.

This stratified approach sets the groundwork for the integrative algorithm described in the following section. To further operationalize the continuum framework, Table 3 summarizes selected biomarkers and clinical proxies that have been associated with differential treatment response across the monoaminergic–glutamatergic spectrum. Although many of these markers remain investigational, they illustrate how biological signals may eventually inform mechanism-based stratification.

Together, these signals illustrate how depressive phenotypes may reflect different positions along a shared neurobiological continuum. This perspective enables a transition from symptom-based categorization toward mechanism-informed treatment sequencing.

### 6.3. Toward an Integrated Clinical Algorithm

Building upon the dimensional heuristics described above, an integrated clinical algorithm can be conceptualized to align therapeutic strategies with the dominant neurobiological axis operating in each patient. This approach moves beyond categorical syndromes to a mechanistic, spectrum-based model that acknowledges depression as a disorder of adaptive network regulation, where monoaminergic signaling and glutamatergic plasticity jointly determine mood, cognition, and resilience [1,13]. In clinical practice, this framework supports a stepwise algorithm grounded in neurobiological plausibility. Patients presenting with preserved cognitive flexibility, intact reward sensitivity, and first-episode depressive syndromes may initially benefit from monoaminergic modulation, such as SSRIs, SNRIs, or multimodal antidepressants, that stabilize neuromodulatory tone and restore network synchrony [4,7]. As treatment progresses, failure to achieve remission or persistence of anhedonia, cognitive rigidity, or emotional blunting suggests a shift toward glutamatergic dysregulation and reduced synaptic adaptability, warranting a transition to plasticity-enhancing interventions such as ketamine, esketamine, or neurosteroid therapies [2,15]. At more advanced stages, where chronicity, neurotrophic depletion, and impaired stress adaptation dominate the clinical picture, the algorithm favors multi-modal or sequential combinations that target both axes, such as augmenting monoaminergic agents with glutamatergic modulators or employing interventions that converge on neuroplastic signaling, including electroconvulsive therapy (ECT) and transcranial magnetic stimulation (TMS).

Emerging evidence suggests that integrative treatments not only normalize neurotransmission but also restore synaptic architecture and connectivity in prefrontal–limbic networks, promoting sustained recovery and reducing relapse risk [16,17]. Importantly, this algorithm is dynamic rather than prescriptive. It emphasizes iterative reassessment of the patient’s biological and phenomenological profile, using clinical response, cognitive flexibility, and neurobiological proxies, such as changes in connectivity or neurotrophic markers, to guide subsequent steps.

The ultimate goal is a precision-based treatment trajectory, where the therapeutic choice reflects the patient’s dominant axis of dysfunction and their residual capacity for plastic adaptation [52,71]. 

Such an approach reframes antidepressant sequencing as a process of functional matching—aligning treatment mechanisms to evolving neurobiological states rather than adhering to uniform categorical steps. Taken together, this framework represents a translational bridge between neurobiology and clinical practice. It provides a structured yet flexible model for guiding treatment selection along mechanistic axes, thereby improving personalization of care and potentially enhancing long-term outcomes. These algorithmic principles mark a transitional step toward data-driven, biologically informed psychiatry, where future clinical decisions may integrate molecular biomarkers, neuroimaging parameters, and computational modeling to predict individual trajectories of recovery and resilience.

### 6.4. Research Priorities

The conceptual shift from monoaminergic imbalance to a dynamic model of adaptive connectivity introduces new priorities for translational research.

While current clinical frameworks still rely heavily on symptom clusters, the integration of biomarkers of plasticity, stress reactivity, and network connectivity could enable a more biologically grounded taxonomy of depressive disorders [12,13].

This approach requires the development of multimodal datasets that combine neuroimaging, peripheral markers (e.g., BDNF, inflammatory cytokines, metabolomics), and cognitive-behavioral measures of adaptability into unified predictive models. Future research should prioritize longitudinal multimodal designs capable of capturing the dynamic trajectories of neuroplastic change, rather than static biomarker snapshots.

Such frameworks could elucidate how fluctuations in synaptic strength, dendritic complexity, and glial–neuronal coupling parallel clinical shifts in motivation, cognition, and affective regulation [72,73]. 

Particular attention should be given to the construct of plasticity reserve, the brain’s intrinsic capacity to mount adaptive responses, which may represent a mechanistic bridge between neurobiology, clinical trajectory, and treatment responsiveness. The next generation of research should also integrate computational psychiatry and artificial intelligence approaches to model these complex relationships. Machine-learning algorithms trained on multimodal inputs could stratify patients based on individualized plasticity signatures, predicting both treatment response and relapse vulnerability [2].

Such predictive modeling would enable truly mechanism-guided precision psychiatry, where interventions are dynamically adjusted as neurobiological parameters evolve over time.

Moreover, a paradigm shift toward brain health rather than brain disease would reframe depressive disorders within a preventive neuroscience perspective, emphasizing early identification of stress-related vulnerability and resilience mechanisms [74]. Ultimately, advancing this research agenda will require integrative collaboration across neurobiology, pharmacology, data science, and clinical practice. Large-scale consortia capable of harmonizing molecular, imaging, and behavioral data are essential to validate biomarkers, operationalize mechanistic constructs, and translate laboratory findings into actionable clinical tools.

## 7. Limitations

Several limitations warrant consideration. First, much of the biomarker evidence remains group-level and cross-sectional, limiting direct clinical applicability at the individual level. Second, inflammatory and connectivity markers demonstrate heterogeneity across cohorts, raising concerns about reproducibility and effect size inflation in early subtyping studies. Third, rapid-acting antidepressant mechanisms in humans are often inferred from convergent translational data rather than direct causal mediation analyses. Finally, while the monoamine–glutamate continuum offers conceptual coherence, its clinical utility depends on prospective biomarker-enriched trials capable of validating mechanism-based treatment matching. It should be noted that many of the mechanistic interpretations discussed in this review are based on converging evidence from preclinical, imaging, and translational studies, and should therefore be interpreted with appropriate caution when applied to individual patients.

## 8. Conclusions

Over the past decades, the understanding of depression has evolved from a model of monoaminergic deficiency to a broader framework centered on neuroplasticity and adaptive connectivity. This shift reflects growing recognition that depressive disorders arise not from isolated neurotransmitter deficits but from failures in the brain’s capacity to reorganize, learn, and maintain functional integration across networks. Within this continuum, monoaminergic regulation ensures affective tone and motivational drive, while glutamatergic plasticity underpins learning, cognitive flexibility, and recovery potential. Their interplay defines a dynamic equilibrium that can be disrupted by stress, neuroinflammation, or metabolic dysregulation, leading to distinct but overlapping depressive phenotypes. Understanding these axes offers a path toward a biologically grounded classification of depression that transcends traditional diagnostic categories. Clinically, this framework supports adaptive, mechanism-based treatment algorithms in which therapeutic choices align with the dominant neurobiological imbalance and the patient’s residual capacity for plastic change. Such an approach enables iterative adjustment over time, moving from monoaminergic modulation to plasticity-enhancing interventions as needed, anchoring clinical decision-making in neurobiological logic rather than syndromic convention. Ultimately, reframing depression as a disorder of impaired adaptive connectivity bridges molecular neuroscience, clinical phenomenology, and therapeutic innovation. This integrated view not only enhances conceptual coherence but also points toward a future psychiatry that is truly precision-oriented, biologically informed, and dynamically responsive to the evolving brain states of each individual.

## Figures and Tables

**Table 1:** Clinical dimensions linked to the monoaminergic axis (evidence-informed signals).

Dimension/Subtype	Key Clinical Features	Putative Biological Anchors/Biomarkers	Treatments with Comparatively Stronger Evidence/Typical Clinical Preference	Representative Study/Finding
**Melancholic**	Psychomotor retardation, anergia, diurnal mood variation	HPA-axis hyperactivity (e.g., cortisol non-suppression); objective psychomotor disturbance (e.g., CORE); reduced reward responsivity	Dual-action antidepressants (e.g., SNRIs) and TCAs often show comparative benefit in more severe/psychomotor-retarded presentations; ECT considered in severe, high-burden cases (context-dependent)	Objective psychomotor disturbance is a discriminant marker for melancholic depression and supports tiered subtyping approaches (Parker et al., 1999) [43]
**Atypical**	Mood reactivity, hypersomnia, hyperphagia, interpersonal rejection sensitivity	Circadian/energy dysregulation; reward/DA-related features; (often) lower HPA-axis activation vs. melancholic	MAOIs (e.g., phenelzine) show comparative efficacy vs. TCAs in atypical features; SSRIs remain commonly effective/used	Meta-analytic evidence supports MAOI superiority vs. TCA and comparable outcomes to SSRIs in atypical depression (Henkel et al., 2006) [44]
**Anxious depression**	Agitation, somatic tension, insomnia, prominent anxiety comorbidity	Heightened limbic reactivity; elevated arousal/stress responsivity; often higher baseline symptom burden	SSRIs/SNRIs are standard first-line; augmentation strategies (e.g., atypical antipsychotics) may be required in non-remitters; short-term anxiolytics may be considered when clinically appropriate	In STAR*D, anxious depression was common and associated with lower remission across sequential treatments (Fava et al., 2008) [45]
**Inflammatory**	Fatigue, anhedonia, psychomotor slowing, cognitive dulling; elevated CRP/IL-6	CRP, IL-6; kynurenine pathway activation (e.g., higher KYN; QA/KA imbalance as research markers)	Baseline inflammation may moderate antidepressant response: comparatively better outcomes reported with noradrenergic/dual-action strategies (e.g., TCAs/NRIs) in higher CRP vs. SSRIs in lower CRP; anti-inflammatory augmentation is investigational/selected cases	CRP acted as a differential predictor of response between escitalopram and nortriptyline in MDD (Uher et al., 2014); mechanistic framing for inflammatory biomarkers as moderators (Hashimoto, 2015) [48,49]
**Seasonal affective disorder (SAD)**	Hypersomnia, hyperphagia, carbohydrate craving; winter recurrence	Photoperiod/circadian dysregulation; serotonergic vulnerability	Light therapy is established; bupropion XL for prevention; SSRIs (e.g., sertraline) effective in acute SAD	Placebo-controlled sertraline trial supports efficacy in SAD (Moscovitch et al., 2004); anticipatory bupropion XL reduced winter relapse risk (Modell et al., 2005) [46,47]

Abbreviations: CRP, C-reactive protein; DA, dopamine; HPA, hypothalamic–pituitary–adrenal; IL-6, interleukin-6; KYN, kynurenine; KA, kynurenic acid; QA, quinolinic acid; MAOI, monoamine oxidase inhibitor; MDD, major depressive disorder; NRI, norepinephrine reuptake inhibitor; SNRI, serotonin–norepinephrine reuptake inhibitor; SSRI, selective serotonin reuptake inhibitor; TCA, tricyclic antidepressant; ECT, electroconvulsive therapy.

**Table 2:** Clinical dimensions linked to the glutamatergic/neuroplasticity axis.

Dimension/Subtype	Key Clinical Features	Putative Biological Anchors/Biomarkers	Treatments with Comparatively Stronger Evidence/Typical Clinical Preference	Representative Study/Finding
Treatment-resistant major depression (TRD)	Nonresponse to ≥2 adequate antidepressant trials; severe symptom burden, suicidality, functional impairment	Reduced “plasticity reserve” (conceptual); network rigidity/connectivity alterations (research measures); EEG gamma changes as engagement markers (research); Glx alterations on 1H-MRS (group-level)	Ketamine/esketamine show rapid antidepressant effects; effects often require maintenance strategies and monitoring; response heterogeneity suggests value of biomarker-enriched stratification	Proof-of-concept RCT showed rapid symptom reduction after IV ketamine vs. placebo (Zarate et al., 2006); connectivity findings support network-level engagement in responders (Abdallah et al., 2015) [15,16]
Bipolar depression	Depressive episodes persisting despite adequate mood stabilizer therapy; high functional impairment	Glutamatergic/circuit dysregulation signatures overlapping TRD (research); careful monitoring for affective switch	Ketamine may produce rapid antidepressant effects as add-on in TR bipolar depression; requires monitoring for dissociation and mood destabilization	Randomized crossover add-on trial reported rapid improvement vs. placebo with transient dissociative effects (Diazgranados et al., 2010) [65]
Post-partum depression (PPD)	Onset within 6 months postpartum; emotional lability, anhedonia; impaired maternal bonding	Neurosteroid withdrawal → altered GABA-A tone; E/I imbalance (mechanistic anchor)	Brexanolone (IV) is effective with rapid and sustained improvement in PPD; (zuranolone can be discussed in text if included elsewhere)	Two phase 3 RCTs: brexanolone produced rapid and sustained HAM-D reduction vs. placebo (Meltzer-Brody et al., 2018) [64]
Severe depression with prominent circuit disruption (late-life; psychotic features)	Severe/psychotic depression in elderly; cognitive slowing, psychomotor retardation; circuit-level disconnection	Macroplasticity/circuit remodeling (conceptual); hippocampal–limbic structural/functional alterations	ECT remains highly effective in severe/psychotic/late-life depression; framed here as a neuroplasticity-engaging intervention (not a “glutamate-specific” drug)	MRI evidence of transient hippocampal volume increase after ECT, dissociable from peripheral BDNF changes (Bouckaert et al., 2016) [66]
Anhedonia/rapid-acting mechanisms (translational)	Core reward-processing deficits and motivational blunting; cognitive rigidity; persistent anhedonia	Reward-circuit dysfunction (e.g., ventral striatum; research); inflammation–dopamine links; glutamatergic throughput deficits (conceptual)	Clinically established rapid-acting options include ketamine/esketamine and dextromethorphan–bupropion (AXS-05); (2R,6R)-HNK is best framed as a mechanistic/translational candidate rather than an established treatment	Ketamine metabolite HNK shows antidepressant-like effects via AMPA-dependent mechanisms in translational work (Zanos et al., 2016); AXS-05 demonstrated efficacy in MDD RCTs (Tabuteau et al., 2022) [14,63]

Abbreviations: ECT, electroconvulsive therapy; EEG, electroencephalography; Glx, glutamate+glutamine; HNK, (2R,6R)-hydroxynorketamine; 1H-MRS, proton magnetic resonance spectroscopy; MDD, major depressive disorder; PPD, postpartum depression; RCT, randomized controlled trial; TRD, treatment-resistant depression.

**Table 3:** Biomarker-informed stratification signals across the monoamine–glutamate continuum.

Biomarker/Proxy	Putative Axis Implication	Type of Evidence	Treatment Signal (Hypothesis-Generating or Emerging)	Key References
CRP (<1 mg/L vs. ≥1 mg/L)	Inflammation–monoamine interaction; serotonergic sensitivity vs. noradrenergic preference	Differential predictor in RCT (moderation analysis)	Lower CRP → comparatively better SSRI response; higher CRP → comparatively better response to noradrenergic/dual-action agents	Uher et al., 2014 [48]; Hashimoto, 2015 [49]
IL-6/TNF-α elevation	Inflammatory subtype; kynurenine activation; dopamine suppression	Observational + mechanistic	Reduced SSRI response likelihood; potential signal for alternative or augmentation strategies	Miller & Raison, 2016 [30]
Kynurenine pathway (QA/KA imbalance)	Glutamatergic overactivation via NMDA agonism (quinolinic acid)	Meta-analytic + translational	Supports inflammatory–glutamatergic subtype hypothesis; treatment matching still investigational	Marx et al., 2021 [54]; Savitz, 2020 [59]
1H-MRS Glx (ACC/PFC)	Cortical hypoglutamatergia; plasticity impairment	Meta-analysis (group-level)	May identify glutamatergic involvement; not yet clinically actionable	Moriguchi et al., 2019 [10]
Resting-state connectivity (e.g., sgACC/DMN patterns)	Network-level dysregulation; plasticity deficit	Subtyping + biomarker research	Connectivity signatures may predict differential response to neuromodulation or rapid-acting agents (research stage)	Drysdale et al., 2017 [18]; Abdallah et al., 2016 [73]
EEG gamma increase post-ketamine	Synaptic potentiation/AMPA engagement	Translational biomarker	Potential pharmacodynamic marker of glutamatergic engagement	Cornwell et al., 2012 [56]
Psychomotor retardation (CORE)	Melancholic subtype; HPA-axis activation	Clinical phenotyping	Supports selection of dual-action/TCAs in severe melancholic presentations (clinical signal)	Parker et al., 1999 [43]
Anhedonia severity (reward-processing deficits)	Dopamine–glutamate interface; reduced plasticity	Clinical + mechanistic	Signal toward plasticity-enhancing or rapid-acting interventions in persistent cases	Felger & Treadway, 2017 [31]; Zanos et al., 2016 [14]

Abbreviations: ACC—Anterior cingulate cortex; CRP—C-reactive protein; DMN—Default mode network; EEG—Electroencephalography; Glx—Combined glutamate and glutamine signal measured by magnetic resonance spectroscopy; HNK—(2R,6R)-Hydroxynorketamine; IL-6—Interleukin-6; KA—Kynurenic acid; KYN—Kynurenine; NMDA—N-methyl-D-aspartate receptor; QA—Quinolinic acid; RCT—Randomized controlled trial; TNF-α—Tumor necrosis factor alpha; 1H-MRS—Proton magnetic resonance spectroscopy. Note: Biomarkers listed are at heterogeneous stages of validation. Most remain research-stage and are not currently standard-of-care decision tools.

## Data Availability

No new data were created or analyzed in this study. Data sharing is not applicable to this article.

## Abbreviations

The following abbreviations are used in this manuscript:

BDNF	Brain-Derived Neurotrophic Factor
DMN	Default Mode Network
IDO	Indoleamine 2,3-dioxygenase
MDD	Major Depressive Disorder
mTOR	Mechanistic Target of Rapamycin
NMDAR	N-Methyl-D-Aspartate Receptor
TRD	Treatment-Resistant Depression

## References

[1] Sanacora G., Treccani G., Popoli M. (2011). Towards a glutamate hypothesis of depression: An emerging frontier of neuropsychopharmacology for mood disorders. Neuropharmacology.

[2] McIntyre R.S., Jain R. (2024). Glutamatergic Modulators for Major Depression from Theory to Clinical Use. CNS Drugs.

[3] Elhwuegi A.S. (2004). Central monoamines and their role in major depression. Prog. Neuro-Psychopharmacol. Biol. Psychiatry.

[4] Delgado P.L. (2000). Depression: The case for a monoamine deficiency. J. Clin. Psychiatry.

[5] Bauer M., Whybrow P.C., Angst J., Versiani M., Möller H.J. (2002). World Federation of Societies Biological Psychiatry Task Force on Treatment Guidelines for Unipolar Depressive Disorders World Federation of Societies of Biological Psychiatry (WFSBP) Guidelines for Biological Treatment of Unipolar Depressive Disorders Part 1: Acute continuation treatment of major depressive disorder. World J. Biol. Psychiatry.

[6] Ruhé H.G., Mason N.S., Schene A.H. (2007). Mood is indirectly related to serotonin, norepinephrine and dopamine levels in humans: A meta-analysis of monoamine depletion studies. Mol. Psychiatry.

[7] Krishnan V., Nestler E.J. (2008). The molecular neurobiology of depression. Nature.

[8] Sanacora G., Zarate C.A., Krystal J.H., Manji H.K. (2008). Targeting the glutamatergic system to develop novel, improved therapeutics for mood disorders. Nat. Rev. Drug Discov..

[9] Henter I.D., Park L.T., Zarate C.A. (2021). Novel Glutamatergic Modulators for the Treatment of Mood Disorders: Current Status. CNS Drugs.

[10] Moriguchi S., Takamiya A., Noda Y., Horita N., Wada M., Tsugawa S., Plitman E., Sano Y., Tarumi R., ElSalhy M. (2019). Glutamatergic neurometabolite levels in major depressive disorder: A systematic review and meta-analysis of proton magnetic resonance spectroscopy studies. Mol. Psychiatry.

[11] Rial D., Lemos C., Pinheiro H., Duarte J.M., Gonçalves F.Q., Real J.I., Prediger R.D., Gonçalves N., Gomes C.A., Canas P.M. (2016). Depression as a Glial-Based Synaptic Dysfunction. Front. Cell Neurosci..

[12] McEwen B.S. (1999). Stress and hippocampal plasticity. Annu. Rev. Neurosci..

[13] Duman R.S., Aghajanian G.K. (2012). Synaptic dysfunction in depression: Potential therapeutic targets. Science.

[14] Zanos P., Moaddel R., Morris P.J., Georgiou P., Fischell J., Elmer G.I., Alkondon M., Yuan P., Pribut H.J., Singh N.S. (2016). NMDAR inhibition-independent antidepressant actions of ketamine metabolites. Nature.

[15] Zarate C.A., Singh J.B., Carlson P.J., Brutsche N.E., Ameli R., Luckenbaugh D.A., Charney D.S., Manji H.K. (2006). A randomized trial of an N-methyl-D-aspartate antagonist in treatment-resistant major depression. Arch. Gen. Psychiatry.

[16] Abdallah C.G., Sanacora G., Duman R.S., Krystal J.H. (2015). Ketamine and rapid-acting antidepressants: A window into a new neurobiology for mood disorder therapeutics. Annu. Rev. Med..

[17] McIntyre R.S., Rosenblat J.D., Nemeroff C.B., Sanacora G., Murrough J.W., Berk M., Brietzke E., Dodd S., Gorwood P., Ho R. (2021). Synthesizing the Evidence for Ketamine and Esketamine in Treatment-Resistant Depression: An International Expert Opinion on the Available Evidence and Implementation. Am. J. Psychiatry.

[18] Drysdale A.T., Grosenick L., Downar J., Dunlop K., Mansouri F., Meng Y., Fetcho R.N., Zebley B., Oathes D.J., Etkin A. (2017). Resting-state connectivity biomarkers define neurophysiological subtypes of depression. Nat. Med..

[19] Cuthbert B.N., Insel T.R. (2013). Toward the future of psychiatric diagnosis: The seven pillars of RDoC. BMC Med..

[20] Kotov R., Krueger R.F., Watson D., Achenbach T.M., Althoff R.R., Bagby R.M., Brown T.A., Carpenter W.T., Caspi A., Clark L.A. (2017). The Hierarchical Taxonomy of Psychopathology (HiTOP): A dimensional alternative to traditional nosologies. J. Abnorm. Psychol..

[21] Gyurak A., Haase C.M., Sze J., Goodkind M.S., Coppola G., Lane J., Miller B.L., Levenson R.W. (2013). The effect of the serotonin transporter (5-HTTLPR) polymorphism on empathic and self-conscious emotional reactivity. Emotion.

[22] Lesch K.P., Bengel D., Heils A., Sabol S.Z., Greenberg B.D., Petri S., Benjamin J., Müller C.R., Hamer D.H., Murphy D.L. (1996). Association of Anxiety-Related Traits with a Polymorphism in the Serotonin Transporter Gene Regulatory Region. Science.

[23] Duman R.S., Voleti B. (2012). Signaling pathways underlying the pathophysiology and treatment of depression: Novel mechanisms for rapid-acting agents. Trends Neurosci..

[24] Castrén E., Monteggia L.M. (2021). Brain-Derived Neurotrophic Factor Signaling in Depression and Antidepressant Action. Biol. Psychiatry.

[25] Spies M., Knudsen G.M., Lanzenberger R., Kasper S. (2015). The serotonin transporter in psychiatric disorders: Insights from PET imaging. Lancet Psychiatry.

[26] Miller J.M., Hesselgrave N., Ogden R.T., Sullivan G.M., Oquendo M.A., Mann J.J., Parsey R.V. (2013). Positron emission tomography quantification of serotonin transporter in suicide attempters with major depressive disorder. Biol. Psychiatry.

[27] Drevets W.C., Price J.L., Furey M.L. (2008). Brain structural and functional abnormalities in mood disorders: Implications for neurocircuitry models of depression. Brain Struct. Funct..

[28] Mayberg H.S., Liotti M., Brannan S.K., McGinnis S., Mahurin R.K., Jerabek P.A., Silva J.A., Tekell J.L., Martin C.C., Lancaster J.L. (1999). Reciprocal limbic-cortical function and negative mood: Converging PET findings in depression and normal sadness. Am. J. Psychiatry.

[29] Pariante C.M., Lightman S.L. (2008). The HPA axis in major depression: Classical theories and new developments. Trends Neurosci..

[30] Miller A.H., Raison C.L. (2016). The role of inflammation in depression: From evolutionary imperative to modern treatment target. Nat. Rev. Immunol..

[31] Felger J.C., Treadway M.T. (2017). Inflammation Effects on Motivation and Motor Activity: Role of Dopamine. Neuropsychopharmacology.

[32] Meyer J.H., Ginovart N., Boovariwala A., Sagrati S., Hussey D., Garcia A., Young T., Praschak-Rieder N., Wilson A.A., Houle S. (2006). Elevated monoamine oxidase a levels in the brain: An explanation for the monoamine imbalance of major depression. Arch. Gen. Psychiatry.

[33] Cipriani A., Furukawa T.A., Salanti G., Chaimani A., Atkinson L.Z., Ogawa Y., Leucht S., Ruhe H.G., Turner E.H., Higgins J.P.T. (2018). Comparative efficacy and acceptability of 21 antidepressant drugs for the acute treatment of adults with major depressive disorder: A systematic review and network meta-analysis. Lancet.

[34] Thase M.E. (2012). The role of monoamine oxidase inhibitors in depression treatment guidelines. J. Clin. Psychiatry.

[35] McGrath P.J., Stewart J.W., Fava M., Trivedi M.H., Wisniewski S.R., Nierenberg A.A., Thase M.E., Davis L., Biggs M.M., Shores-Wilson K. (2006). Tranylcypromine Versus Venlafaxine Plus Mirtazapine Following Three Failed Antidepressant Medication Trials for Depression: A STAR*D Report. Am. J. Psychiatry.

[36] McIntyre R.S., Lophaven S., Olsen C.K. (2014). A randomized, double-blind, placebo-controlled study of vortioxetine on cognitive function in depressed adults. Int. J. Neuropsychopharmacol..

[37] Watanabe N., Omori I.M., Nakagawa A., Cipriani A., Barbui C., Churchill R., Furukawa T.A. (2011). Mirtazapine versus other antidepressive agents for depression. Cochrane Database Syst. Rev..

[38] Baldessarini R.J., Tondo L., Hennen J. (2003). Lithium treatment and suicide risk in major affective disorders: Update and new findings. J. Clin. Psychiatry.

[39] Joffe R.T., Sokolov S.T., Singer W. (1995). Thyroid hormone treatment of depression. Thyroid.

[40] Nelson J.C., Papakostas G.I. (2009). Atypical antipsychotic augmentation in major depressive disorder: A meta-analysis of placebo-controlled randomized trials. Am. J. Psychiatry.

[41] Godlewska B.R., Norbury R., Selvaraj S., Cowen P.J., Harmer C.J. (2012). Short-term SSRI treatment normalises amygdala hyperactivity in depressed patients. Psychol. Med..

[42] Meyer J.H., Wilson A.A., Sagrati S., Hussey D., Carella A., Potter W.Z., Ginovart N., Spencer E.P., Cheok A., Houle S. (2004). Serotonin Transporter Occupancy of Five Selective Serotonin Reuptake Inhibitors at Different Doses: An [11C]DASB Positron Emission Tomography Study. Am. J. Psychiatry.

[43] Parker G., Wilhelm K., Mitchell P., Roy K., Hadzi-Pavlovic D. (1999). Subtyping depression: Testing algorithms and identification of a tiered model. J. Nerv. Ment. Dis..

[44] Henkel V., Mergl R., Allgaier A.K., Kohnen R., Möller H.J., Hegerl U. (2006). Treatment of depression with atypical features: A meta-analytic approach. Psychiatry Res..

[45] Fava M., Rush A.J., Alpert J.E., Balasubramani G.K., Wisniewski S.R., Carmin C.N., Biggs M.M., Zisook S., Leuchter A., Howland R. (2008). Difference in treatment outcome in outpatients with anxious versus nonanxious depression: A STAR*D report. Am. J. Psychiatry.

[46] Modell J.G., Rosenthal N.E., Harriett A.E., Krishen A., Asgharian A., Foster V.J., Metz A., Rockett C.B., Wightman D.S. (2005). Seasonal Affective Disorder and Its Prevention by Anticipatory Treatment with Bupropion XL. Biol. Psychiatry.

[47] Moscovitch A., Blashko C.A., Eagles J.M., Darcourt G., Thompson C., Kasper S., Lane R.M. (2004). A placebo-controlled study of sertraline in the treatment of outpatients with seasonal affective disorder. Psychopharmacology.

[48] Uher R., Tansey K.E., Dew T., Maier W., Mors O., Hauser J., Dernovsek M.Z., Henigsberg N., Souery D., Farmer A. (2014). An inflammatory biomarker as a differential predictor of outcome of depression treatment with escitalopram and nortriptyline. Am. J. Psychiatry.

[49] Hashimoto K. (2015). Inflammatory biomarkers as differential predictors of antidepressant response. Int. J. Mol. Sci..

[50] Williams L.M., Rush A.J., Koslow S.H., Wisniewski S.R., Cooper N.J., Nemeroff C.B., Schatzberg A.F., Gordon E. (2011). International Study to Predict Optimized Treatment for Depression (iSPOT-D), a randomized clinical trial: Rationale and protocol. Trials.

[51] Choudary P.V., Molnar M., Evans S.J., Tomita H., Li J.Z., Vawter M.P., Myers R.M., Bunney W.E., Akil H., Watson S.J. (2005). Altered cortical glutamatergic and GABAergic signal transmission with glial involvement in depression. Proc. Natl. Acad. Sci. USA.

[52] Duman R.S., Li N. (2012). A neurotrophic hypothesis of depression: Role of synaptogenesis in the actions of NMDA receptor antagonists. Philos. Trans. R. Soc. Lond. B Biol. Sci..

[53] Li N., Lee B., Liu R.J., Banasr M., Dwyer J.M., Iwata M., Li X.Y., Aghajanian G., Duman R.S. (2010). mTOR-dependent synapse formation underlies the rapid antidepressant effects of NMDA antagonists. Science.

[54] Marx W., McGuinness A.J., Rocks T., Ruusunen A., Cleminson J., Walker A.J., Gomes-da-Costa S., Lane M., Sanches M., Diaz A.P. (2021). The kynurenine pathway in major depressive disorder, bipolar disorder, and schizophrenia: A meta-analysis of 101 studies. Mol. Psychiatry.

[55] Sheline Y.I., Price J.L., Yan Z., Mintun M.A. (2010). Resting-state functional MRI in depression unmasks increased connectivity between networks via the dorsal nexus. Proc. Natl. Acad. Sci. USA.

[56] Cornwell B.R., Salvadore G., Furey M., Marquardt C.A., Brutsche N.E., Grillon C., Zarate C.A. (2012). Synaptic potentiation is critical for rapid antidepressant response to ketamine in treatment-resistant major depression. Biol. Psychiatry.

[57] Rajkowska G., Stockmeier C.A. (2013). Astrocyte pathology in major depressive disorder: Insights from human postmortem brain tissue. Curr. Drug Targets.

[58] Haroon E., Miller A.H., Sanacora G. (2017). Inflammation, Glutamate, and Glia: A Trio of Trouble in Mood Disorders. Neuropsychopharmacology.

[59] Savitz J. (2020). The Kynurenine Pathway: A Finger in Every Pie. Mol. Psychiatry.

[60] Newport D.J., Carpenter L.L., McDonald W.M., Potash J.B., Tohen M., Nemeroff C.B., APA Council of Research Task Force on Novel Biomarkers and Treatments (2015). Ketamine and Other NMDA Antagonists: Early Clinical Trials and Possible Mechanisms in Depression. Am. J. Psychiatry.

[61] Daly E.J., Singh J.B., Fedgchin M., Cooper K., Lim P., Shelton R.C., Thase M.E., Winokur A., Van Nueten L., Manji H. (2018). Efficacy and Safety of Intranasal Esketamine Adjunctive to Oral Antidepressant Therapy in Treatment-Resistant Depression: A Randomized Clinical Trial. JAMA Psychiatry.

[62] Jeon H.J., Ju P.C., Sulaiman A.H., Aziz S.A., Paik J.W., Tan W., Bai D., Li C.T. (2022). Long-term Safety and Efficacy of Esketamine Nasal Spray Plus an Oral Antidepressant in Patients with Treatment-resistant Depression- an Asian Sub-group Analysis from the SUSTAIN-2 Study. Clin. Psychopharmacol. Neurosci..

[63] Tabuteau H., Jones A., Anderson A., Jacobson M., Iosifescu D.V. (2022). Effect of AXS-05 (Dextromethorphan-Bupropion) in Major Depressive Disorder: A Randomized Double-Blind Controlled Trial. Am. J. Psychiatry.

[64] Meltzer-Brody S., Colquhoun H., Riesenberg R., Epperson C.N., Deligiannidis K.M., Rubinow D.R., Li H., Sankoh A.J., Clemson C., Schacterle A. (2018). Brexanolone injection in post-partum depression: Two multicentre, double-blind, randomised, placebo-controlled, phase 3 trials. Lancet.

[65] Diazgranados N., Ibrahim L., Brutsche N.E., Newberg A., Kronstein P., Khalife S., Kammerer W.A., Quezado Z., Luckenbaugh D.A., Salvadore G. (2010). A Randomized Add-on Trial of an N-methyl-d-aspartate Antagonist in Treatment-Resistant Bipolar Depression. Arch. Gen. Psychiatry.

[66] Bouckaert F., Dols A., Emsell L., De Winter F.L., Vansteelandt K., Claes L., Sunaert S., Stek M., Sienaert P., Vandenbulcke M. (2016). Relationship Between Hippocampal Volume, Serum BDNF, and Depression Severity Following Electroconvulsive Therapy in Late-Life Depression. Neuropsychopharmacology.

[67] SCHILDKRAUTJJ (1965). The catecholamine hypothesis of affective disorders: A review of supporting evidence. Am. J. Psychiatry.

[68] Coppen A. (1967). The Biochemistry of Affective Disorders. Br. J. Psychiatry.

[69] Duman R.S., Sanacora G., Krystal J.H. (2019). Altered connectivity in depression: GABA and glutamate neurotransmitter deficits and reversal by novel treatments. Neuron.

[70] Ohashi S., Matsumoto M., Togashi H., Ueno K., Yoshioka M. (2003). The serotonergic modulation of synaptic plasticity in the rat hippocampo-medial prefrontal cortex pathway. Neurosci. Lett..

[71] Grace A.A. (2016). Dysregulation of the dopamine system in the pathophysiology of schizophrenia and depression. Nat. Rev. Neurosci..

[72] Kavalali E.T., Monteggia L.M. (2012). Synaptic Mechanisms Underlying Rapid Antidepressant Action of Ketamine. Am. J. Psychiatry.

[73] Abdallah C.G., Averill L.A., Collins K.A., Geha P., Schwartz J., Averill C., DeWilde K.E., Wong E., Anticevic A., Tang C.Y. (2016). Ketamine Treatment and Global Brain Connectivity in Major Depression. Neuropsychopharmacology.

[74] Insel T.R. (2009). Disruptive insights in psychiatry: Transforming a clinical discipline. J. Clin. Investig..

